# Diaqua­bis­(*N*,*N*-diethyl­nicotinamide-κ*N* ^1^)bis­(4-formyl­benzoato-κ*O* ^1^)zinc

**DOI:** 10.1107/S1600536812031200

**Published:** 2012-07-14

**Authors:** Mustafa Sertçelik, Nagihan Çaylak Delibaş, Hacali Necefoğlu, Tuncer Hökelek

**Affiliations:** a Department of Chemistry, Kafkas University, 36100 Kars, Turkey; b Department of Physics, Sakarya University, 54187 Esentepe, Sakarya, Turkey; c Department of Physics, Hacettepe University, 06800 Beytepe, Ankara, Turkey

## Abstract

In the title complex, [Zn(C_8_H_5_O_3_)_2_(C_10_H_14_N_2_O)_2_(H_2_O)_2_], the Zn^II^ cation is located on an inversion center and is coordinated by two 4-formyl­benzoate anions, two *N*,*N*-diethyl­nicotinamide (DENA) ligands and two water mol­ecules. The four O atoms in the equatorial plane around the Zn^II^ cation form a slightly distorted square-planar arrangement, while the slightly distorted octa­hedral coordination is completed by the two N atoms of the DENA ligands in the axial positions. The dihedral angle between the carboxyl­ate group and the adjacent benzene ring is 2.96 (11)°, while the pyridine ring and the benzene ring are oriented at a dihedral angle of 79.26 (4)°. The coordinating water mol­ecule links with the carboxyl­ate group *via* an intra­molecular O—H⋯O hydrogen bond. In the crystal, O—H⋯O and weak C—H⋯O hydrogen bonds link the mol­ecules into a three-dimensional supra­molecular network. A π–π contact between the parallel pyridine rings of adjacent mol­ecules may further stabilize the crystal structure [centroid–centroid distance = 3.5654 (8) Å].

## Related literature

For literature on niacin, see: Krishnamachari (1974 ▶). For information on the nicotinic acid derivative *N*,*N*-diethyl­nicotinamide, see: Bigoli *et al.* (1972 ▶). For related structures, see: Aydın *et al.* (2012 ▶); Hökelek *et al.* (1996 ▶, 2009*a* ▶,*b* ▶); Hökelek & Necefoğlu (2007 ▶, 1998 ▶); Necefoğlu, Özbek *et al.* (2011 ▶); Necefoğlu, Maracı *et al.* (2011 ▶); Sertçelik *et al.* (2012 ▶). For bond-length data, see: Allen *et al.* (1987 ▶).
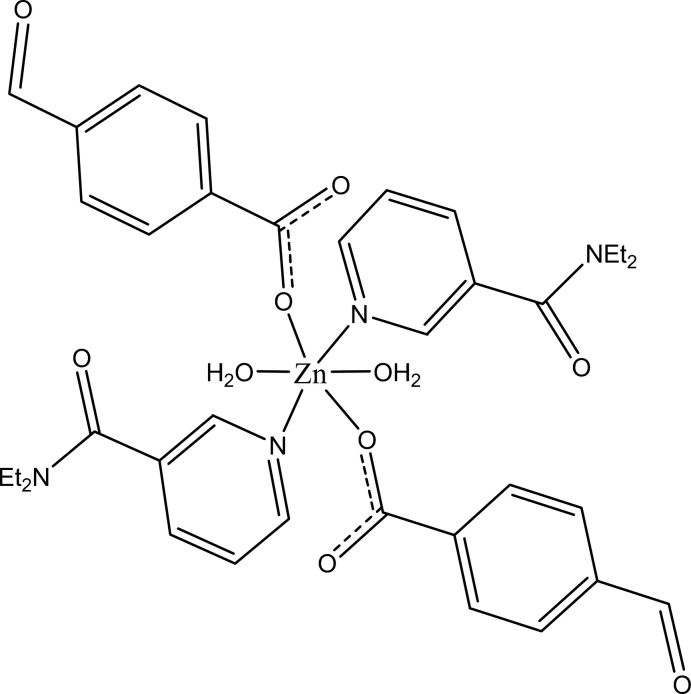


## Experimental

### 

#### Crystal data

[Zn(C_8_H_5_O_3_)_2_(C_10_H_14_N_2_O)_2_(H_2_O)_2_]
*M* *_r_* = 756.13
Triclinic, 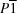

*a* = 7.1988 (2) Å
*b* = 8.5347 (2) Å
*c* = 15.9719 (4) Å
α = 85.435 (3)°
β = 78.010 (3)°
γ = 67.846 (2)°
*V* = 889.03 (4) Å^3^
*Z* = 1
Mo *K*α radiation
μ = 0.75 mm^−1^
*T* = 100 K
0.27 × 0.24 × 0.21 mm

#### Data collection

Bruker Kappa APEXII CCD area-detector diffractometer
Absorption correction: multi-scan (*SADABS*; Bruker, 2005 ▶) *T*_min_ = 0.816, *T*_max_ = 0.854
16164 measured reflections
4409 independent reflections
4128 reflections with *I* > 2σ(*I*)
*R*_int_ = 0.022

#### Refinement

*R*[*F* ^2^ > 2σ(*F* ^2^)] = 0.025
*wR*(*F* ^2^) = 0.081
*S* = 1.16
4409 reflections
246 parameters
H atoms treated by a mixture of independent and constrained refinement
Δρ_max_ = 0.46 e Å^−3^
Δρ_min_ = −0.33 e Å^−3^

### 

Data collection: *APEX2* (Bruker, 2007 ▶); cell refinement: *SAINT* (Bruker, 2007 ▶); data reduction: *SAINT*; program(s) used to solve structure: *SHELXS97* (Sheldrick, 2008 ▶); program(s) used to refine structure: *SHELXL97* (Sheldrick, 2008 ▶); molecular graphics: *ORTEP-3 for Windows* (Farrugia, 1997 ▶); software used to prepare material for publication: *WinGX* (Farrugia, 1999 ▶) and *PLATON* (Spek, 2009 ▶).

## Supplementary Material

Crystal structure: contains datablock(s) I, global. DOI: 10.1107/S1600536812031200/xu5586sup1.cif

Structure factors: contains datablock(s) I. DOI: 10.1107/S1600536812031200/xu5586Isup2.hkl

Additional supplementary materials:  crystallographic information; 3D view; checkCIF report

## Figures and Tables

**Table 1:** Hydrogen-bond geometry (Å, °)

*D*—H⋯*A*	*D*—H	H⋯*A*	*D*⋯*A*	*D*—H⋯*A*
O5—H51⋯O4^i^	0.80 (2)	1.97 (2)	2.7591 (15)	169 (2)
O5—H52⋯O1^ii^	0.85 (2)	1.81 (2)	2.6494 (14)	166 (2)
C4—H4⋯O1^iii^	0.93	2.36	3.1975 (19)	150
C7—H7⋯O3^iv^	0.93	2.60	3.406 (2)	145
C11—H11⋯O1^v^	0.93	2.40	3.3068 (17)	166

Symmetry codes: (i) 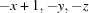; (ii) 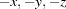; (iii) 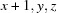; (iv) 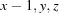; (v) 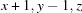.

**Fig. 1.**
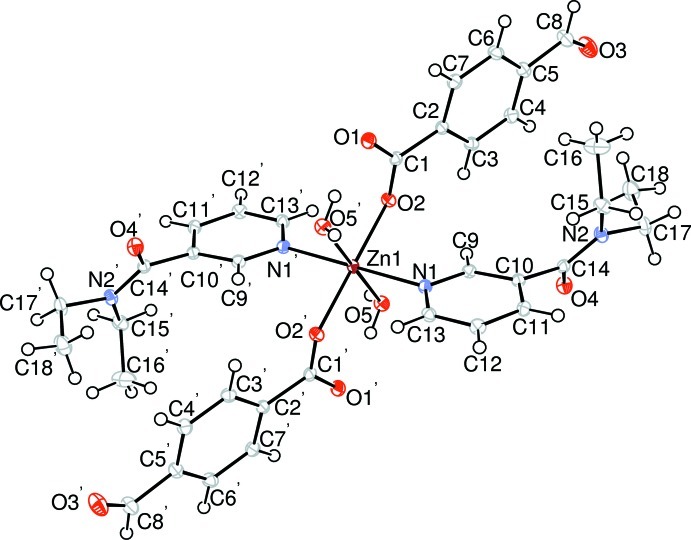
The molecular structure of the title molecule with the atom-numbering scheme. Displacement ellipsoids are drawn at the 50% probability level [symmetry code: (') -x, -y, -z].

## supplementary crystallographic information

### Comment

As a part of our ongoing investigations of transition metal complexes of nicotinamide (NA), one form of niacin (Krishnamachari, 1974), and/or the nicotinic acid derivative *N*,*N*-diethylnicotinamide (DENA), an important respiratory stimulant (Bigoli *et al.*, 1972), the title compound was synthesized and its crystal structure is reported herein.

In the title mononuclear complex, Zn^II^ cation is located on an inversion center and is coordinated by two 4-formylbenzoate (FB) anions, two *N*,*N*-diethylnicotinamide (DENA) ligands and two water molecules, all ligands coordinating in a monodentate manner (Fig. 1). The crystal structures of similar complexes of Cu^II^, Co^II^, Ni^II^, Mn^II^ and Zn^II^ ions, [Cu(C_7_H_5_O_2_)_2_(C_10_H_14_N_2_O)_2_] (Hökelek *et al.*, 1996), [Cu(C_7_H_4_BrO_2_)_2_(C_6_H_6_N_2_O)_2_(H_2_O)_2_] (Necefoğlu, Özbek *et al.*, 2011), [Co(C_6_H_6_N_2_O)_2_(C_7_H_4_NO_4_)_2_(H_2_O)_2_] (Hökelek & Necefoğlu, 1998), [Co(C_9_H_9_O_2_)_2_(C_10_H_14_N_2_O)_2_(H_2_O)_2_] (Necefoğlu, Maracı *et al.*, 2011), [Co(C_7_H_4_IO_2_)_2_(C_6_H_6_N_2_O)_2_(H_2_O)_2_] (Aydın *et al.*, 2012), [Ni(C_7_H_4_ClO_2_)_2_(C_6_H_6_N_2_O)_2_(H_2_O)_2_] (Hökelek *et al.*, 2009*a*), [Ni(C_5_H_5_O_3_)_2_(C_6_H_6_N_2_O)_2_(H_2_O)_2_] (Sertçelik *et al.*, 2012), [Mn(C_9_H_10_NO_2_)_2_(H_2_O)_4_].2H_2_O (Hökelek & Necefoğlu, 2007) and [Zn(C_7_H_4_BrO_2_)_2_(C_6_H_6_N_2_O)_2_(H_2_O)_2_] (Hökelek *et al.*, 2009*b*) have also been reported. In the copper(II) complex mentioned above the two benzoate ions coordinate to the Cu^II^ atom as bidentate ligands, while in the other structures all the ligands coordinate in a monodentate manner.

In the title complex, the four symmetry related O atoms (O2, O2', O5 and O5') in the equatorial plane around the Zn^II^ ion form a slightly distorted square-planar arrangement, while the slightly distorted octahedral coordination is completed by the two symmetry related N atoms of the DENA ligands (N1 and N1') in the axial positions. The near equalities of the C1—O1 [1.2533 (16) Å] and C1—O2 [1.2623 (16) Å] bonds in the carboxylate group indicate delocalized bonding arrangement, rather than localized single and double bonds. The Zn—O bond lengths are 2.1128 (9) Å (for benzoate oxygens) and 2.1289 (10) Å (for water oxygens), and the Cu—N bond length is 2.1452 (11) Å, close to standard values (Allen *et al.*, 1987). The Zn atom is displaced out of the mean-plane of the carboxylate group (O1/C1/O2) by 0.8455 (1) Å. The dihedral angle between the planar carboxylate group and the adjacent benzene ring A (C2—C7) is 2.96 (11)°. The benzene A (C2—C7) and the pyridine B (N1/C9—C13) rings are oriented at a dihedral angle of A/B = 79.26 (4)°. The coordinating water molecule links with the carboxylate group *via* an O—H···O hydrogen bond (Table 1).

In the crystal, intermolecular O—H···O and weak C—H···O hydrogen bonds (Table 1) link the molecules into a three-dimensional supramolecular network, in which they may be effective in the stabilization of the structure. The π–π contact between the pyridine rings, Cg2—Cg2^i^ [symmetry code: (i) 1 - x, 1 - y, -z, where Cg2 is the centroid of the ring B (N1/C9-C13)] may further stabilize the structure, with centroid-centroid distance of 3.5654 (8) Å].

### Experimental

The title compound was prepared by the reaction of ZnSO_4_.H_2_O (0.90 g, 5 mmol) in H_2_O (30 ml) and DENA (1.78 g, 10 mmol) in H_2_O (10 ml) with sodium 4-formylbenzoate (1.72 g, 10 mmol) in H_2_O (100 ml) at room temperature. The mixture was filtered and set aside to crystallize at ambient temperature for several days, giving colorless single crystals.

### Refinement

Atoms H8 (for CH) and H51 and H52 (for H_2_O) were located in a difference Fourier map and were refined freely. The C-bound H-atoms were positioned geometrically with C—H = 0.93, 0.97 and 0.96 Å, for aromatic, methylene and methyl H-atoms, respectively, and constrained to ride on their parent atoms, with *U*_iso_(H) = k × *U*_eq_(C), where k = 1.5 for methyl H-atoms and k = 1.2 for all other H-atoms.

### Crystal data

**Table:**

[Zn(C_8_H_5_O_3_)_2_(C_10_H_14_N_2_O)_2_(H_2_O)_2_]	*Z* = 1
*M**_r_* = 756.13	*F*(000) = 396
Triclinic, *P*1	*D*_x_ = 1.412 Mg m^−^^3^
Hall symbol: -P 1	Mo *K*α radiation, λ = 0.71073 Å
*a* = 7.1988 (2) Å	Cell parameters from 9726 reflections
*b* = 8.5347 (2) Å	θ = 2.6–28.3°
*c* = 15.9719 (4) Å	µ = 0.75 mm^−^^1^
α = 85.435 (3)°	*T* = 100 K
β = 78.010 (3)°	Block, colorless
γ = 67.846 (2)°	0.27 × 0.24 × 0.21 mm
*V* = 889.03 (4) Å^3^	

### Data collection

**Table:**

Bruker Kappa APEXII CCD area-detector diffractometer	4409 independent reflections
Radiation source: fine-focus sealed tube	4128 reflections with *I* > 2σ(*I*)
Graphite monochromator	*R*_int_ = 0.022
φ and ω scans	θ_max_ = 28.3°, θ_min_ = 1.3°
Absorption correction: multi-scan (*SADABS*; Bruker, 2005)	*h* = −9→9
*T*_min_ = 0.816, *T*_max_ = 0.854	*k* = −10→11
16164 measured reflections	*l* = −21→21

### Refinement

**Table:**

Refinement on *F*^2^	Primary atom site location: structure-invariant direct methods
Least-squares matrix: full	Secondary atom site location: difference Fourier map
*R*[*F*^2^ > 2σ(*F*^2^)] = 0.025	Hydrogen site location: inferred from neighbouring sites
*wR*(*F*^2^) = 0.081	H atoms treated by a mixture of independent and constrained refinement
*S* = 1.16	*w* = 1/[σ^2^(*F*_o_^2^) + (0.0442*P*)^2^ + 0.2368*P*] where *P* = (*F*_o_^2^ + 2*F*_c_^2^)/3
4409 reflections	(Δ/σ)_max_ < 0.001
246 parameters	Δρ_max_ = 0.46 e Å^−^^3^
0 restraints	Δρ_min_ = −0.33 e Å^−^^3^

### Special details

**Table:**

Geometry. All esds (except the esd in the dihedral angle between two l.s. planes) are estimated using the full covariance matrix. The cell esds are taken into account individually in the estimation of esds in distances, angles and torsion angles; correlations between esds in cell parameters are only used when they are defined by crystal symmetry. An approximate (isotropic) treatment of cell esds is used for estimating esds involving l.s. planes.
Refinement. Refinement of F^2^ against ALL reflections. The weighted R-factor wR and goodness of fit S are based on F^2^, conventional R-factors R are based on F, with F set to zero for negative F^2^. The threshold expression of F^2^ > 2sigma(F^2^) is used only for calculating R-factors(gt) etc. and is not relevant to the choice of reflections for refinement. R-factors based on F^2^ are statistically about twice as large as those based on F, and R- factors based on ALL data will be even larger.

### Fractional atomic coordinates and isotropic or equivalent isotropic displacement parameters (Å^2^)

**Table:**

	*x*	*y*	*z*	*U*_iso_*/*U*_eq_	
Zn1	0.0000	0.0000	0.0000	0.01115 (7)	
O1	−0.25853 (15)	0.12741 (13)	0.19910 (6)	0.0168 (2)	
O2	0.02518 (14)	0.11856 (12)	0.10604 (6)	0.01404 (19)	
O3	0.44445 (18)	0.18906 (16)	0.45766 (7)	0.0274 (2)	
O4	0.73443 (15)	−0.33358 (13)	0.12604 (6)	0.0173 (2)	
O5	0.28172 (15)	0.01887 (14)	−0.06344 (6)	0.01568 (19)	
H51	0.282 (3)	0.112 (3)	−0.0758 (13)	0.027 (5)*	
H52	0.295 (3)	−0.030 (3)	−0.1102 (15)	0.037 (6)*	
N1	0.18033 (16)	−0.23838 (14)	0.04858 (7)	0.0129 (2)	
N2	0.61125 (17)	−0.42797 (14)	0.25277 (7)	0.0148 (2)	
C1	−0.0800 (2)	0.12606 (16)	0.18050 (8)	0.0128 (2)	
C2	0.02005 (19)	0.13218 (16)	0.25409 (8)	0.0128 (2)	
C3	0.2155 (2)	0.13794 (17)	0.23866 (8)	0.0142 (2)	
H3	0.2864	0.1348	0.1827	0.017*	
C4	0.3048 (2)	0.14827 (17)	0.30598 (8)	0.0157 (3)	
H4	0.4346	0.1529	0.2954	0.019*	
C5	0.1978 (2)	0.15163 (17)	0.38982 (8)	0.0158 (3)	
C6	0.0046 (2)	0.14231 (17)	0.40557 (8)	0.0164 (3)	
H6	−0.0650	0.1429	0.4616	0.020*	
C7	−0.0845 (2)	0.13219 (17)	0.33800 (8)	0.0150 (3)	
H7	−0.2133	0.1254	0.3487	0.018*	
C8	0.2874 (2)	0.1654 (2)	0.46331 (9)	0.0211 (3)	
H8	0.198 (3)	0.153 (2)	0.5225 (11)	0.016 (4)*	
C9	0.32059 (19)	−0.24272 (16)	0.09356 (8)	0.0127 (2)	
H9	0.3407	−0.1432	0.1000	0.015*	
C10	0.43636 (19)	−0.38948 (16)	0.13075 (8)	0.0125 (2)	
C11	0.4100 (2)	−0.53987 (17)	0.11979 (8)	0.0141 (2)	
H11	0.4850	−0.6401	0.1444	0.017*	
C12	0.2696 (2)	−0.53643 (17)	0.07137 (8)	0.0151 (3)	
H12	0.2503	−0.6349	0.0620	0.018*	
C13	0.1585 (2)	−0.38386 (17)	0.03717 (8)	0.0139 (2)	
H13	0.0643	−0.3823	0.0048	0.017*	
C14	0.6039 (2)	−0.38178 (16)	0.17105 (8)	0.0130 (2)	
C15	0.4557 (2)	−0.47566 (18)	0.31161 (9)	0.0182 (3)	
H15A	0.5213	−0.5862	0.3356	0.022*	
H15B	0.3592	−0.4845	0.2797	0.022*	
C16	0.3398 (3)	−0.3501 (2)	0.38430 (11)	0.0314 (4)	
H16A	0.2364	−0.3849	0.4192	0.047*	
H16B	0.2768	−0.2398	0.3610	0.047*	
H16C	0.4330	−0.3463	0.4185	0.047*	
C17	0.7881 (2)	−0.42804 (18)	0.28570 (9)	0.0186 (3)	
H17A	0.9113	−0.4800	0.2435	0.022*	
H17B	0.8000	−0.4970	0.3371	0.022*	
C18	0.7743 (3)	−0.2522 (2)	0.30647 (10)	0.0243 (3)	
H18A	0.8950	−0.2611	0.3262	0.036*	
H18B	0.6563	−0.2018	0.3503	0.036*	
H18C	0.7627	−0.1830	0.2560	0.036*	

### Atomic displacement parameters (Å^2^)

**Table:**

	*U*^11^	*U*^22^	*U*^33^	*U*^12^	*U*^13^	*U*^23^
Zn1	0.01120 (11)	0.01159 (11)	0.01099 (11)	−0.00424 (8)	−0.00333 (7)	0.00145 (7)
O1	0.0131 (4)	0.0207 (5)	0.0165 (4)	−0.0058 (4)	−0.0033 (3)	−0.0007 (4)
O2	0.0156 (4)	0.0149 (5)	0.0123 (4)	−0.0065 (4)	−0.0029 (3)	0.0005 (3)
O3	0.0292 (6)	0.0390 (7)	0.0226 (5)	−0.0199 (5)	−0.0102 (4)	0.0023 (5)
O4	0.0159 (5)	0.0197 (5)	0.0190 (5)	−0.0096 (4)	−0.0041 (4)	0.0028 (4)
O5	0.0165 (5)	0.0164 (5)	0.0161 (5)	−0.0082 (4)	−0.0034 (4)	0.0005 (4)
N1	0.0124 (5)	0.0132 (5)	0.0126 (5)	−0.0043 (4)	−0.0025 (4)	0.0004 (4)
N2	0.0164 (5)	0.0150 (5)	0.0156 (5)	−0.0073 (4)	−0.0064 (4)	0.0012 (4)
C1	0.0144 (6)	0.0086 (6)	0.0148 (6)	−0.0028 (4)	−0.0045 (5)	0.0006 (4)
C2	0.0148 (6)	0.0097 (6)	0.0134 (6)	−0.0035 (5)	−0.0038 (5)	0.0001 (4)
C3	0.0149 (6)	0.0143 (6)	0.0132 (6)	−0.0058 (5)	−0.0013 (5)	−0.0002 (5)
C4	0.0151 (6)	0.0164 (6)	0.0165 (6)	−0.0067 (5)	−0.0035 (5)	0.0006 (5)
C5	0.0188 (6)	0.0152 (6)	0.0148 (6)	−0.0069 (5)	−0.0053 (5)	0.0008 (5)
C6	0.0183 (6)	0.0176 (7)	0.0121 (6)	−0.0065 (5)	−0.0012 (5)	0.0004 (5)
C7	0.0134 (6)	0.0151 (6)	0.0164 (6)	−0.0054 (5)	−0.0024 (5)	0.0003 (5)
C8	0.0237 (7)	0.0267 (8)	0.0154 (6)	−0.0111 (6)	−0.0059 (5)	0.0008 (5)
C9	0.0137 (6)	0.0116 (6)	0.0136 (5)	−0.0053 (5)	−0.0027 (4)	−0.0007 (4)
C10	0.0113 (5)	0.0142 (6)	0.0114 (5)	−0.0044 (5)	−0.0015 (4)	0.0000 (4)
C11	0.0147 (6)	0.0126 (6)	0.0146 (6)	−0.0041 (5)	−0.0037 (5)	0.0012 (5)
C12	0.0170 (6)	0.0135 (6)	0.0168 (6)	−0.0077 (5)	−0.0035 (5)	−0.0001 (5)
C13	0.0133 (6)	0.0163 (6)	0.0131 (6)	−0.0064 (5)	−0.0029 (4)	−0.0002 (5)
C14	0.0137 (6)	0.0098 (6)	0.0149 (6)	−0.0031 (5)	−0.0039 (5)	−0.0007 (4)
C15	0.0219 (7)	0.0198 (7)	0.0145 (6)	−0.0099 (5)	−0.0035 (5)	0.0015 (5)
C16	0.0314 (9)	0.0381 (10)	0.0255 (8)	−0.0166 (7)	0.0045 (6)	−0.0117 (7)
C17	0.0208 (7)	0.0174 (7)	0.0216 (7)	−0.0076 (5)	−0.0126 (5)	0.0031 (5)
C18	0.0308 (8)	0.0215 (7)	0.0273 (7)	−0.0128 (6)	−0.0145 (6)	0.0010 (6)

### Geometric parameters (Å, º)

**Table:**

Zn1—O2	2.1128 (9)	C7—C2	1.3940 (18)
Zn1—O2^i^	2.1128 (9)	C7—H7	0.9300
Zn1—O5	2.1289 (10)	C8—C5	1.4834 (19)
Zn1—O5^i^	2.1289 (10)	C8—H8	1.049 (17)
Zn1—N1	2.1452 (11)	C9—C10	1.3843 (18)
Zn1—N1^i^	2.1452 (11)	C9—H9	0.9300
O1—C1	1.2533 (16)	C11—C10	1.3954 (18)
O2—C1	1.2623 (16)	C11—C12	1.3857 (18)
O3—C8	1.2057 (19)	C11—H11	0.9300
O4—C14	1.2382 (17)	C12—H12	0.9300
O5—H51	0.81 (2)	C13—C12	1.3843 (19)
O5—H52	0.85 (2)	C13—H13	0.9300
N1—C9	1.3439 (16)	C14—C10	1.5040 (18)
N1—C13	1.3397 (17)	C15—C16	1.521 (2)
N2—C14	1.3390 (17)	C15—H15A	0.9700
N2—C15	1.4655 (18)	C15—H15B	0.9700
N2—C17	1.4747 (17)	C16—H16A	0.9600
C1—C2	1.5143 (17)	C16—H16B	0.9600
C3—C2	1.3958 (18)	C16—H16C	0.9600
C3—C4	1.3866 (18)	C17—C18	1.525 (2)
C3—H3	0.9300	C17—H17A	0.9700
C4—C5	1.3957 (19)	C17—H17B	0.9700
C4—H4	0.9300	C18—H18A	0.9600
C6—C5	1.3926 (19)	C18—H18B	0.9600
C6—C7	1.3885 (19)	C18—H18C	0.9600
C6—H6	0.9300		
			
O2—Zn1—O2^i^	180.00 (8)	O3—C8—C5	125.06 (14)
O2—Zn1—O5	87.26 (4)	O3—C8—H8	122.2 (10)
O2^i^—Zn1—O5	92.74 (4)	C5—C8—H8	112.7 (10)
O2—Zn1—O5^i^	92.74 (4)	N1—C9—C10	122.57 (12)
O2^i^—Zn1—O5^i^	87.26 (4)	N1—C9—H9	118.7
O2—Zn1—N1	88.47 (4)	C10—C9—H9	118.7
O2^i^—Zn1—N1	91.53 (4)	C9—C10—C11	118.95 (12)
O2—Zn1—N1^i^	91.53 (4)	C9—C10—C14	117.31 (12)
O2^i^—Zn1—N1^i^	88.47 (4)	C11—C10—C14	123.23 (11)
O5—Zn1—O5^i^	180.00 (8)	C10—C11—H11	120.8
O5—Zn1—N1	86.62 (4)	C12—C11—C10	118.50 (12)
O5^i^—Zn1—N1	93.38 (4)	C12—C11—H11	120.8
O5—Zn1—N1^i^	93.38 (4)	C11—C12—H12	120.6
O5^i^—Zn1—N1^i^	86.62 (4)	C13—C12—C11	118.86 (12)
N1^i^—Zn1—N1	180.00 (10)	C13—C12—H12	120.6
C1—O2—Zn1	125.27 (9)	N1—C13—C12	123.03 (12)
Zn1—O5—H51	117.4 (14)	N1—C13—H13	118.5
Zn1—O5—H52	98.3 (15)	C12—C13—H13	118.5
H52—O5—H51	107 (2)	O4—C14—N2	121.65 (12)
C9—N1—Zn1	118.79 (9)	O4—C14—C10	117.88 (12)
C13—N1—Zn1	123.15 (9)	N2—C14—C10	120.47 (12)
C13—N1—C9	118.05 (11)	N2—C15—C16	113.09 (12)
C14—N2—C15	124.84 (11)	N2—C15—H15A	109.0
C14—N2—C17	117.10 (12)	N2—C15—H15B	109.0
C15—N2—C17	118.06 (11)	C16—C15—H15A	109.0
O1—C1—O2	126.07 (12)	C16—C15—H15B	109.0
O1—C1—C2	117.12 (11)	H15A—C15—H15B	107.8
O2—C1—C2	116.81 (11)	C15—C16—H16A	109.5
C3—C2—C1	120.65 (11)	C15—C16—H16B	109.5
C7—C2—C1	119.54 (12)	C15—C16—H16C	109.5
C7—C2—C3	119.81 (12)	H16A—C16—H16B	109.5
C2—C3—H3	119.7	H16A—C16—H16C	109.5
C4—C3—C2	120.65 (12)	H16B—C16—H16C	109.5
C4—C3—H3	119.7	N2—C17—C18	113.79 (12)
C3—C4—C5	119.31 (13)	N2—C17—H17A	108.8
C3—C4—H4	120.3	N2—C17—H17B	108.8
C5—C4—H4	120.3	C18—C17—H17A	108.8
C4—C5—C8	120.74 (13)	C18—C17—H17B	108.8
C6—C5—C4	120.23 (12)	H17A—C17—H17B	107.7
C6—C5—C8	119.03 (12)	C17—C18—H18A	109.5
C5—C6—H6	119.8	C17—C18—H18B	109.5
C7—C6—C5	120.31 (12)	C17—C18—H18C	109.5
C7—C6—H6	119.8	H18A—C18—H18B	109.5
C2—C7—H7	120.2	H18A—C18—H18C	109.5
C6—C7—C2	119.67 (12)	H18B—C18—H18C	109.5
C6—C7—H7	120.2		
			
O5—Zn1—O2—C1	−166.52 (10)	O1—C1—C2—C3	−177.42 (12)
O5^i^—Zn1—O2—C1	13.48 (10)	O1—C1—C2—C7	2.52 (18)
N1—Zn1—O2—C1	−79.84 (10)	O2—C1—C2—C3	3.18 (18)
N1^i^—Zn1—O2—C1	100.16 (10)	O2—C1—C2—C7	−176.87 (12)
O2—Zn1—N1—C9	−30.48 (10)	C4—C3—C2—C1	178.20 (12)
O2^i^—Zn1—N1—C9	149.52 (10)	C4—C3—C2—C7	−1.7 (2)
O2—Zn1—N1—C13	148.70 (10)	C2—C3—C4—C5	0.5 (2)
O2^i^—Zn1—N1—C13	−31.30 (10)	C3—C4—C5—C6	0.9 (2)
O5—Zn1—N1—C9	56.87 (10)	C3—C4—C5—C8	−179.00 (13)
O5^i^—Zn1—N1—C9	−123.13 (10)	C7—C6—C5—C4	−1.0 (2)
O5—Zn1—N1—C13	−123.96 (10)	C7—C6—C5—C8	178.92 (13)
O5^i^—Zn1—N1—C13	56.04 (10)	C5—C6—C7—C2	−0.3 (2)
Zn1—O2—C1—O1	−29.35 (18)	C6—C7—C2—C1	−178.30 (12)
Zn1—O2—C1—C2	149.99 (9)	C6—C7—C2—C3	1.6 (2)
Zn1—N1—C9—C10	176.76 (9)	O3—C8—C5—C4	6.7 (2)
C13—N1—C9—C10	−2.46 (19)	O3—C8—C5—C6	−173.20 (15)
Zn1—N1—C13—C12	−177.44 (10)	N1—C9—C10—C11	1.38 (19)
C9—N1—C13—C12	1.74 (19)	N1—C9—C10—C14	173.43 (12)
C15—N2—C14—O4	175.78 (12)	C12—C11—C10—C9	0.49 (19)
C15—N2—C14—C10	−5.06 (19)	C12—C11—C10—C14	−171.08 (12)
C17—N2—C14—O4	−3.29 (19)	C10—C11—C12—C13	−1.16 (19)
C17—N2—C14—C10	175.87 (11)	N1—C13—C12—C11	0.1 (2)
C14—N2—C15—C16	−112.92 (15)	O4—C14—C10—C9	−55.69 (17)
C17—N2—C15—C16	66.14 (17)	O4—C14—C10—C11	116.00 (14)
C14—N2—C17—C18	77.07 (16)	N2—C14—C10—C9	125.13 (13)
C15—N2—C17—C18	−102.06 (14)	N2—C14—C10—C11	−63.18 (18)

Symmetry code: (i) −*x*, −*y*, −*z*.

### Hydrogen-bond geometry (Å, º)

**Table:**

*D*—H···*A*	*D*—H	H···*A*	*D*···*A*	*D*—H···*A*
O5—H51···O4^ii^	0.80 (2)	1.97 (2)	2.7591 (15)	169 (2)
O5—H52···O1^i^	0.85 (2)	1.81 (2)	2.6494 (14)	166 (2)
C4—H4···O1^iii^	0.93	2.36	3.1975 (19)	150
C7—H7···O3^iv^	0.93	2.60	3.406 (2)	145
C11—H11···O1^v^	0.93	2.40	3.3068 (17)	166

Symmetry codes: (i) −*x*, −*y*, −*z*; (ii) −*x*+1, −*y*, −*z*; (iii) *x*+1, *y*, *z*; (iv) *x*−1, *y*, *z*; (v) *x*+1, *y*−1, *z*.
